# Cell wall composition throughout development for the model grass *Brachypodium distachyon*

**DOI:** 10.3389/fpls.2012.00266

**Published:** 2012-12-05

**Authors:** David M. Rancour, Jane M. Marita, Ronald D. Hatfield

**Affiliations:** Cell Wall Biology and Utilization Unit, U.S. Dairy Forage Research Center, Agriculture Research Service, U.S. Department of AgricultureMadison, WI, USA

**Keywords:** plant cell wall, biomass, *Brachypodium distachyon*, grass, chemical composition

## Abstract

Temperate perennial grasses are important worldwide as a livestock nutritive energy source and a potential feedstock for lignocellulosic biofuel production. The annual temperate grass *Brachypodium distachyon* has been championed as a useful model system to facilitate biological research in agriculturally important temperate forage grasses based on phylogenetic relationships. To physically corroborate genetic predictions, we determined the chemical composition profiles of organ-specific cell walls throughout the development of two common diploid accessions of *Brachypodium distachyon*, Bd21-3 and Bd21. Chemical analysis was performed on cell walls isolated from distinct organs (i.e., leaves, sheaths, stems, and roots) at three developmental stages of (1) 12-day seedling, (2) vegetative-to-reproductive transition, and (3) mature seed fill. In addition, we have included cell wall analysis of embryonic callus used for genetic transformations. Composition of cell walls based on components lignin, hydroxycinnamates, uronosyls, neutral sugars, and protein suggests that *Brachypodium distachyon* is similar chemically to agriculturally important forage grasses. There were modest compositional differences in hydroxycinnamate profiles between accessions Bd21-3 and Bd21. In addition, when compared to agronomical important C3 grasses, more mature *Brachypodium* stem cell walls have a relative increase in glucose of 48% and a decrease in lignin of 36%. Though differences exist between *Brachypodium* and agronomical important C3 grasses, *Brachypodium distachyon* should be still a useful model system for genetic manipulation of cell wall composition to determine the impact upon functional characteristics such as rumen digestibility or energy conversion efficiency for bioenergy production.

## INTRODUCTION

Grasses are important economically worldwide as a nutritive energy source for ruminant livestock. Furthermore, grasses have been viewed as a highly attractive feedstock for second-generation lignocellulosic biofuel production (Vogel, 2008; Naik et al., 2010), given recent fossil fuels cost increases, the prospect of limited future petroleum availability, and the negative environmental impact of fossil fuel use; thus alternative fuel replacements need to be identified. The increasing world population will place increased burden on current agriculture practices to ensure adequate food and fuel for future generations (Food and Agriculture Organization of the United Nations [FAO], 2011). Therefore, steps taken to improve agricultural output abundance and quality are needed.

The power of appropriate biological research model systems cannot be underestimated. For example, the advances made in plant research through the use of the model dicot *Arabidopsis thaliana* have had widespread influence on our understanding over all of plant biology ranging from fundamental molecular pathways to broader implications into multicellular organism development (Koornneef and Meinke, 2010). However, clear distinctions exist between monocots and dicots and therefore, a model system for monocots is prudent to have and utilize.

Recent work has championed *Brachypodium distachyon*, false brome, as a functional genomics model system for grasses and cereals (Draper et al., 2001; Garvin, 2007; Garvin et al., 2008; Opanowicz et al., 2008; Vogel and Bragg, 2009; Bevan et al., 2010; Brkljacic et al., 2011; Mur et al., 2011; Philippe, 2011). *Brachypodium distachyon* is a temperate annual grass that has many features ideal for a model system including fast growth with small stature, a small genome size with a high degree of synteny with other grass genomes, and diploid accessions to facilitate Mendelian genetic studies. The genome of the diploid accession Bd21 was recently sequenced (International Brachypodium Initiative, 2010) and adds to a growing list of functional genomic tools and resources available for this plant (Huo et al., 2006; Garvin et al., 2008; Gu et al., 2009; Mockler et al., 2010; Cao et al., 2011; Mur et al., 2011; Thole et al., 2012). Included in its utility is the potential for genetic manipulation through *Agrobacterium*-mediated T-DNA transformation (Christiansen et al., 2005; Pãcurar, 2008; Vain et al., 2008; Vogel and Hill, 2008; Alves et al., 2009; Thole et al., 2009, 2012). Therefore, in principle, the stage is set for significant progress in understanding fundamental molecular, cellular, and developmental processes unique to grass biology.

The proposal that *Brachypodium distachyon* is a model for forage grass cell wall biology and biomass production has been made primarily though phylogenetic comparisons (Bevan et al., 2010). Primary cell wall characterization has been performed through analyzing either early seedling development (Christensen et al., 2010) or endosperm maturation during seed development (Guillon et al., 2011, 2012; Opanowicz et al., 2011) but developmentally mature secondary cell wall containing material has not been evaluated. The general chemical components of monocot cell walls include structural carbohydrates, lignin and phenolics, proteins, and hydrophobics such as waxes, cutins, and suberins (Carpita, 1996). It is documented that the relative proportion and molecular variation within these chemical subgroups vary within a single species throughout its development (Carpita, 1996; Fincher, 2009) and significantly between themselves in various forage grass species (Hatfield et al., 2009). From a practical perspective, forage grasses, when used as feeds or as potential feedstock for biofuels, are used at advanced stages of development to supply sufficient yields for downstream applications. Therefore, plant material at comparable developmental maturity needs to be analyzed to allow for direct evaluation to determine whether *Brachypodium distachyon* cell walls and biomass are chemically in line with commonly used forage grass cell walls and biomass. In addition, a detailed cell wall composition map for *Brachypodium distachyon* would be useful in establishing a chemical framework from which results of genetic manipulations of cell wall biosynthesis can be evaluated.

To allow a comparison of cell wall composition between *Brachypodium distachyon* and agriculturally important C3 forage grasses, we undertook the chemical characterization of cell walls prepared from four plant organs at three distinct developmental maturities of the diploid *Brachypodium distachyon* accession Bd21-3. The plant developmental maturities used correspond to Biologische **B**undesanstalt, **B**undessortenamt und **CH**emische Industrie (BBCH)-scale developmental stages of (1) expanding inflorescence/heading (BBCH stage range 57–61; “expanding”) and (2) seed fill (BBCH stage range 69–75; “mature”; Hack et al., 1992; Hong et al., 2011). Cell wall analyses were performed on (3) 12-day-old seedlings to complement earlier studies (Christensen et al., 2010). In addition, full analysis was performed on embryonic callus used for transformations (Vogel and Hill, 2008). Analysis of cell walls from organs of a second broadly used *Brachypodium* accession, Bd21, was performed for the two more mature developmental stages to assess possible chemical variation with Bd21-3. From developing plants, the plant parts used for cell wall isolations included leaves, sheaths, stems, and, in the case of seedlings, roots to assess organ-specific variations in composition. For each plant organ and developmental stage, lignin, hydroxycinnamates, uronosyls, neutral sugars, and protein were quantified. This work provides the first complete plant-developmental map of cell wall composition in *Brachypodium distachyon* and supports utilizing *Brachypodium* cell wall composition as an appropriate model for forage grass cell wall studies.

## MATERIALS AND METHODS

### MATERIALS

*Brachypodium distachyon* seed was kindly obtained from Dr. John Vogel (accession Bd21-3) and Dr. David Garvin (accession Bd21). Chemicals used were of high grade and obtained from Sigma-Aldrich, Fisher Scientific, Acros, and Fluka.

### PLANT GROWTH

All *Brachypodium distachyon* seeds were surface sterilized with 10% bleach/0.1% TX-100 and rinsed four times with sterile water. Sterilized seed were incubated for 5 days in sterile water at 4°C. For expanding and mature developmental staged growth, sterile seed were aseptically plated between two pieces of autoclaved Whatman 3M filter paper wetted with sterile dH_2_O in a Petri dish, sealed with parafilm, and incubated in a Percival Scientific (Perry, IA, USA) growth chamber under conditions to promote germination (24°C, 20 h fluorescent light with an average photon flux of 120 μmol s^−1^ m^−2^). After 7 days, germinated seeds were transplanted to 4″ circular pots containing soil mix (2:1 v/v Premier PromixBX:Happy Frog Potting Soil with Mycorrhizae and Humic Acid; Premier Horticulture, Quakertown, PA, USA and FoxFarm Soil & Fertilizer Co., Arcata, CA, USA, respectively) and transferred to a Conviron (Pembina, ND, USA) E15 growth chamber maintained at 20 h light, 24°C, and an average cool white fluorescent light photon flux of 180 μmol s^−1^ m^−2^. After 12 days, plants were transferred to a greenhouse maintained at 25–35°C, supplemented with Na-lamp light for a total average photon flux of 270 μmol s^−1^ m^−2^ for a 20-h light cycle, and grown to either of the two most mature developmental stages for tissue collection. The two mature developmental stages correspond to the BBCH-scale developmental stages of expanding inflorescence/heading (BBCH stage range 57–61) and seed fill (BBCH stage range 69–75; Hack et al., 1992; Hong et al., 2011). Under our growth conditions, heading occurred after approximately 3 weeks and seed fill after 4.5–5 weeks in the greenhouse. Therefore day totals from germination to harvest were approximately 40 and 54 days, respectively. Plant parts were harvested by hand using razor blades for cutting and pooled according to plant part type. The plant parts harvested included: (1) leaf blades (broken along the ligule/auricle interface), (2) sheaths (from ligule/auricle interface to stem collar), (3) stems (with all sheath and reproductive organ material removed), and (4) all reproductive organs and contents within at the mature stage. Harvested material was quickly frozen in liquid nitrogen and stored at−70°C. For both the expanding and mature developmental stages, two biological replicates of each accession were harvested and analyzed separately.

To obtain *Brachypodium* seedling organs, surface sterilized seed were aseptically plated onto a galvanized steel mesh support (~2 mm size square hole) in a 25 cm × 38 cm glass pan (above 0.5× strength liquid MS growth medium; Young, 2001). The system was capped with an inverted second glass pan of the same size and sealed with 3M surgical tape. The whole system was wrapped in aluminum foil and incubated at 4°C. After 5 days, the growth system was transferred to a growth chamber to support seedling growth (24°C, 16 h fluorescent light with an average photon flux of 120 μmol s^−1^ m^−2^). Seeds were allowed to germinate and seedlings were grown for 12 days. Seedling organs of (1) leaf blades, (2) sheath/stem, and (3) roots were harvested by hand dissection, frozen in liquid nitrogen, and stored at−70°C. Only seedling organs from Bd21-3 were analyzed. Plant parts from three independent growth/harvest cycles were pooled for compiled, individual cell wall preparations for each organ.

Embryonic callus from Bd21-3 was generated according to the method used in the published protocol for *Brachypodium distachyon* transformations (Vogel and Hill, 2008). In brief, Bd21-3 immature embryos were aseptically dissected from surface sterilized seed. Embryos were plated onto solid callus induction medium (CIM) in 20 mm × 100 mm Petri dishes, sealed with parafilm, and incubated at 28°C in the dark for 3 weeks. Callus tissue fragments (2–4 mm) were plated onto fresh CIM and allowed to grow for 2 weeks at 28°C in the dark. Callus was split and transferred once more to fresh medium and allowed to grow for 3 weeks prior to harvest. Growth medium-free calli were harvested directly into a 50-ml conical tube containing liquid nitrogen and stored at−70°C.

All harvested organs and tissue was homogenized frozen using a Spex SamplePrep Freezer/Mill (Model 6870; Metuchen, NJ, USA). Homogenization cycle parameters used were: 10 min pre-cool, three cycles of 2 min homogenization at 10 bps with 1 min cool down intermissions. All plant sample powders were kept frozen and stored at−70°C.

### CELL WALL PREPARATIONS

Starch-free cell wall preparations were made based on the procedures of Hatfield et al. (2009). In brief, frozen plant sample powders (~5 g) were weighed directly into pre-weighed, dry Oakridge centrifuge tubes in which all treatments and extractions took place. Samples were extracted with 50 mM NaCl overnight at 4°C followed by 30 min at 40°C the next morning. Material was pelleted by centrifugation at 32,900 × *g* (at average radius) for 20 min at 20°C. Supernatants were decanted and pellets extracted two times more with 50 mM NaCl for 30 min at 40°C. Pelleted material was suspended in 50 mM Tris-acetate pH 6.0 and heated for 2 h in boiling water bath for starch denaturation. Samples were cooled (~22°C), supplemented with 40 U of amyloglucosidase (Fluka BioChemika) and 20 U of 1,4-α-D-glucan glucanohydrolase (α-amylase; Sigma-Aldrich, St. Louis, MO, USA) and incubated at 55°C for 2 h with shaking. Reactions were terminated by adding ethanol (95%) to a final concentration of 80% and mixed at room temperature for 30 min. Samples were centrifuged as above, supernatants decanted away and EtOH extracts properly disposed. Pellets were extracted an additional three times with 80% ethanol. Acetone was added to the final 80% ethanol extracted pellet and samples were stored overnight at 4°C. The next day the samples were brought to room temperature and incubated with shaking for 30 min. Material was centrifuged as before and supernatants removed. Subsequently, pellets were extracted one time with chloroform:methanol (1:1 v/v) and then four times with acetone with all extractions involving incubations of 30 min at room temperature with shaking. Final cell wall residues were air dried in a fume hood to remove organic solvents, heated to 55°C overnight to fully dry, and weighed to determine yields. Cell wall material was stored dry at room temperature until further use.

### ACETYL BROMIDE LIGNIN

Acetyl bromide (AcBr) lignin determination was performed essentially according to Hatfield et al. (1999a). Cell wall samples (~25 mg) were analyzed in duplicate for each preparation. A positive control sample of maize (post-anthesis) stock cell wall was analyzed in parallel. Dry samples were incubated 2 h at 50°C in 2.5 ml 25% (v/v) AcBr in glacial acetic acid. Samples were cooled to room temperature and 1.5 ml of sample was cleared by centrifugation (3 min, 12,000 × *g*) in a microfuge. Clarified supernatant (0.5 ml) was transferred to a glass vial containing 9.5 ml of 0.42 M NaOH, 18.4 mM hydroxylamine, and 12.4 M acetic acid. Absorbance scans from 350 to 250 nm were performed. The absorbance at 280 nm was used to calculate sample lignin content. The extinction coefficients used for calculations were 18.126 and 17.747 g^−1^ l cm^−1^, respectively for *Brachypodium distachyon* cell wall samples and maize stock cell wall standard (Fukushima and Hatfield, 2004). The extinction coefficient used for *Brachypodium* samples was the average of values determined for C3 grasses obtained from purified HCl–dioxane lignin preparations (Fukushima and Hatfield, 2004). Cell wall preparations were analyzed in duplicate and data were compiled according to plant accession, developmental stage, and organ type based on two biological replicates. For seedling organs and callus, error in data represents the standard deviation of analytical replicates.

### ESTER- AND ETHER-LINKED PHENOLICS

The sequential analysis of cell wall ester- and ether-linked phenolic moieties was performed as described (Hatfield et al., 2009). Approximately 70 mg of dried cell wall material per sample was used for analysis. Ferulic acid dimers (diferulic acid, DFA) presented represent the sum of all forms detected, which include: 8-8′-DFA (aryl tetralin), 8-8′-DFA, 8-5′-DFA. 8-O-4′-DFA, 8-5′-DFA (benzofuran), 5-5′-DFA, 8-5′-DFA (decarboxylated), and 4-O-5′-DFA. Ester- and ether-linked phenolics were identified and quantified as trimethylsilane derivatives (40 μl TMSI, Pierce and 10 μl pyridine) by GLC-FID (HP6890) on a ZB-1 column (Phenomenex, Torrance, CA; Zebron 100% dimethypolysiloxane; 30 m × 0.25 mm, 0.25 μm film). The GLC conditions were injector 315°C, detector 300°C, and a temperature program of 220°C 1 min, 4°C min^−1^ to 248°C held 1 min, followed by 30°C min^−1^ to 300°C before holding for 16 min. All GC temperature programs were run at 20 psi constant pressure and split ratio 35:1. Cell wall preparations were analyzed in duplicate and data was compiled according to plant accession, developmental stage, and organ type based on two biological replicates. For seedling organs and callus, error in data represents the standard deviation of analytical replicates.

### NEUTRAL SUGAR ANALYSIS

Analysis of cell wall carbohydrate content was performed based on the Saeman hydrolysis (Saeman et al., 1963) as modified by Hatfield et al. (2009). In brief, cell wall samples (~25 mg) and neutral sugar standards were analyzed in parallel and incubated in 0.5 ml 12 M sulfuric acid (cold at addition) for 2 h at room temperature (~22°C), diluted with 3.5 ml dH_2_O followed by further incubation for 3 h at 100°C. After cooling partially, samples were centrifuged at ~200 × *g* for 10 min to pellet insoluble material. Two hundred microliters of particulate-free supernatant from each were transferred to test tubes containing 1.8 ml dH_2_O and used for uronosyl analysis (see below). To each remaining hydrolysate, an inositol internal standard was added (2.5 mg sample^−1^, 100 μl of a 25-mg ml^−1^ solution in dH_2_O), 1 ml of spiked sample was diluted with 10 ml dH_2_O, and samples were neutralized with solid BaCO_3_. Samples were cleared of precipitant by centrifugation (5 min, room temperature, 1500 × *g*) and the supernatants filtered (1 μm glass fiber membrane, Acrodisc 25 mm syringe filter, Pall Life Sciences) into clean tubes and dried. Sugars were converted to alditol acetate derivatives according to the procedure of Blakeney et al. (1982) and identified and quantified by GLC-FID on a Shimadzu GC-2010 using a 007-225 (50% cyanopropylphenyl) methylpolysiloxane column (Catalog #007-225, 30 m × 0.25 mm with 0.25 μm film thickness, Quadrex Corporation, Woodbridge, CT, USA). The GLC conditions were injector 220°C, detector 240°C, and a temperature program of 215°C for 2 min, 4°C min^−1^ to 230°C before holding for 11.25 min run at constant linear velocity of 33.4 cm s^−1^ and split ratio 25:1. Cell wall preparations were analyzed in duplicate and data was compiled according to plant accession, developmental stage, and organ type based on two biological replicates. For seedling organs and callus, error in data represents the standard deviation of analytical replicates.

### URONOSYL ANALYSIS

Uronosyl content of cell wall samples was performed according to Blumenkrantz and Asboe-Hansen (Blumenkrantz and Asboe-Hansen, 1973). Diluted and clarified supernatant samples from sulfuric acid hydrolysis (see above) were used as inputs for analysis. All analytical samples were run in duplicate of two independent cell wall aliquots per cell wall preparation. For each analytical sample, a matching background control that did not get the color reagent, 3-phenylphenol, was run in parallel. A standard curve was generated using galacturonic acid (GalA) dissolved in dH_2_O. Two hundred microliters of diluted sulfuric acid supernatants, GalA dissolved in dH_2_O, or pure dH_2_O were added to glass test tubes and chilled on ice. To the test tubes, 1.2 ml 12.5 mM sodium tetraborate in 18 M sulfuric acid was added and the mixed samples were heated for 5 min at 100°C followed by cooling in an ice water bath. For color generation, 20 μl of 0.15% 3-phenylphenol in 0.5% NaOH was mixed in and samples were incubated a minimum of 30 min prior to reading the absorbance at 520 nm. The absorbance for water-only, no 3-phenylphenol samples were used to 0 the spectrophotometer. The absorbance of samples without color reagent added served as background values for their partner samples and was subtracted prior to calculation uronosyl concentrations. Standard curves of GalA were linear over a concentration range 0–100 μg ml^−1^ with *R*^2^ > 0.98.

### PROTEIN

Crude cell wall protein content was analyzed using a Vario Max CN macroelemental combustion analyzer (Elementar Americas, Mt. Laurel, NJ, USA) to determine sample nitrogen content. Approximately 50 mg of cell wall sample was used for analysis. Glutamic acid was used as a standard. Crude protein was calculated as the percent nitrogen times a factor of 6.25, according to manufacturer’s protocols.

### CALCULATIONS AND STATISTICS

All mathematical calculations were performed using Excel 2011 for Mac (Microsoft Corp., Redmond WA, USA). Statistical analyses were performed using Prism 5 for Mac OS X (GraphPad Software, La Jolla, CA, USA). All statistical comparisons were performed using one-way ANOVA with a *post hoc* Tukey test. An alpha of 0.05 was used to evaluate the significance of the comparison. All comparisons described are statistically significant unless indicated.

## RESULTS

To determine if *Brachypodium distachyon* could be a practical model system for forage grass research, we characterized the cell wall compositions of organs derived from the diploid accessions Bd21-3 and Bd21. The accessions Bd21-3 and Bd21, both used for transformations but with claimed differences in transformability (Vogel and Hill, 2008; Alves et al., 2009), were analyzed to determine if there were potential changes in cell wall characteristics that may influence their transformation properties. Our analyses utilized methods frequently used for forage analysis to allow for direct comparison of results between agronomical important forage grasses and the putative model, *Brachypodium distachyon*.

**FIGURE 1:**
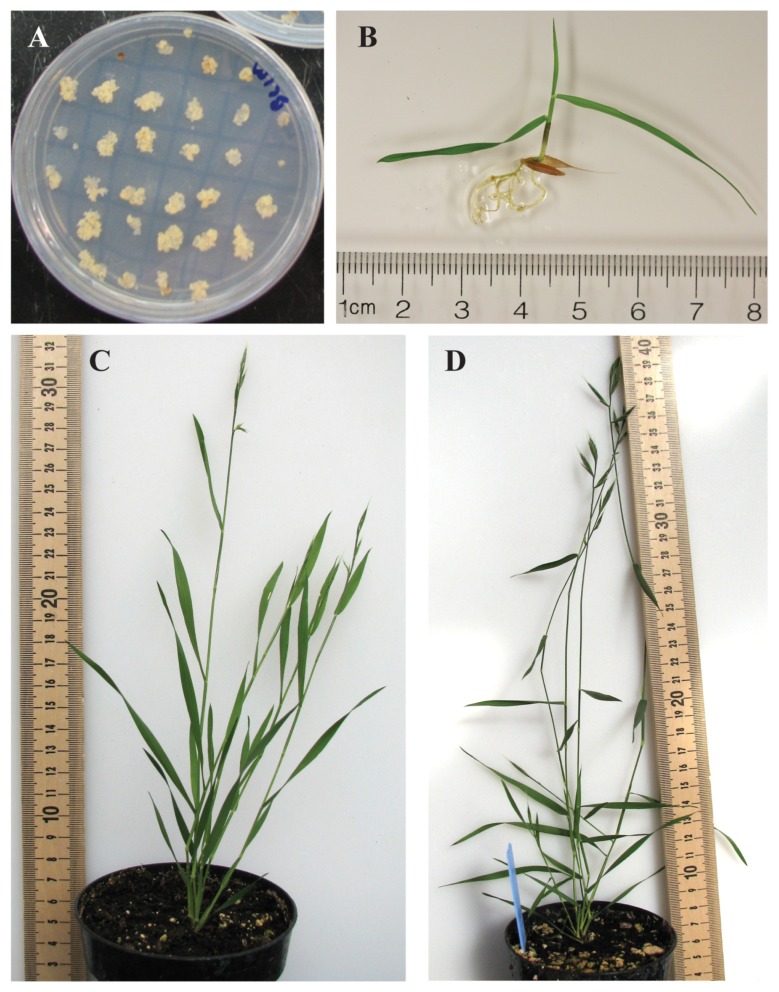
***Brachypodium distachyon* developmental stages used for generation of tissue-specific cell walls for chemical analysis**. **(A)** Embryonic callus. **(B)** Twelve-day-old seedling. **(C)** Expanding inflorescence/heading stage (BBCH stage range 57–61). **(D)** Seed fill/mature stage (BBCH stage range 69–75). All measurement scales are in centimeters. All samples shown are the Bd21-3 accession.

Grass cell wall composition changes with developmental maturity and organ type (MacAdam et al., 1996; Morrison et al., 1998; MacAdam and Grabber, 2002; Abedon et al., 2006; Jung and Casler, 2006). We analyzed organ types related to distinct developmental stages of plant growth to assess what changes occur in *Brachypodium* during its development and to use this as a baseline for future work in the genetic manipulation of grass cell wall biosynthesis. The developmental stages analyzed represent a series including (1) young seedlings, a staple of researchers studying early plant development and primary cell wall compositions, (2) the elongating stem/inflorescence spike stage, the transition to reproductive growth, and (3) the mature reproductive plant at seed fill stage (**Figures 1B–D**). The latter two developmental stages represent stages of growth when grasses are typically harvested as forage and for ensiling. In addition, we chose to analyze cell walls from embryonic callus used in plant transformation procedures for comparison (**Figure 1A**).

Cell wall yields as a proportion of fresh weight for the isolated organs varied (**Table 1**). The lowest yields were observed with seedling and callus tissue. A general trend was observed where organs from more mature plants resulted in higher cell wall weight yields. Relative yield differences most likely can be attributed to water content of the respective sample. All subsequent chemical analyses were performed using individual cell wall preparations from the specified plant samples in order to minimize possible cell wall batch-to-batch variation. Data generated for all expanding and mature samples represents means of two independent biological replicates.

**Table 1:** Average cell wall yields from developing *Brachypodium distachyon* organs.

Developmental stage	Accession	Organ	Average cell wall yield (weight % of input)
Mature	Bd21-3	Leaves	13.2
		Sheath	18.8
		Stems	15.7
		Flower/seeds	21.7
	Bd21	Leaves	14.4
		Sheath	19.6
		Stems	19.1
		Flower/seeds	22.0
Expanding	Bd21-3	Leaves	12.6
		Sheath	15.3
		Stems	10.3
	Bd21	Leaves	13.3
		Sheath	16.9
		Stems	13.6
Seedling	Bd21-3	Leaves	9.1
		Sheath/stem	6
		Roots	2.8
Embryonic	Bd21-3	Callus	2.9

### LIGNIN

**FIGURE 2:**
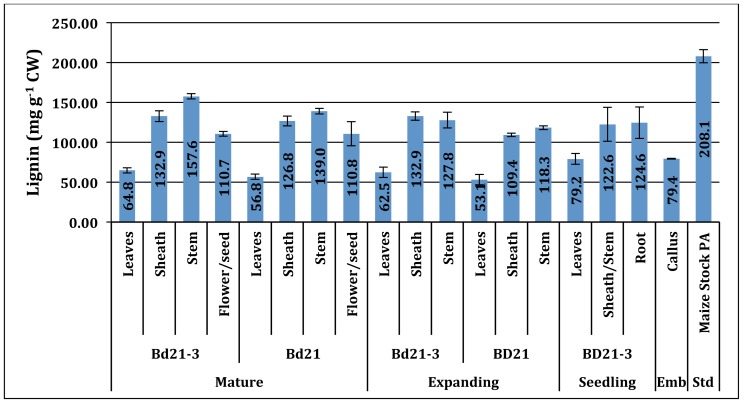
**Acetyl bromide lignin (mg g^−1^ CW) of developing *Brachypodium *cell walls across developmental stage, accession, and tissue**. Std, positive assay standard; Emb, embryonic. Error bars represent the standard deviation from two independent tissue harvests.

Lignin, a complex heteropolymer of hydroxycinnamyl alcohol subunits involved in maintaining cell wall integrity (Boerjan et al., 2003; Vanholme et al., 2010), was analyzed using the AcBr method (Hatfield et al., 1999a; Fukushima and Hatfield, 2004; **Figure 2**).

Lignin content values ranged from 53.1 to 157.6 mg lignin g^−1^ cell wall (CW). The *Brachypodium* organ content of lignin varied primarily based on plant organ source and, to a lesser extent, developmental stage. Typically, leaves had the lowest and sheath and stem had increasingly higher percentages of their walls as lignin with each being significantly different. Reproductive organ tissue lignin levels were comparable to sheath. Surprisingly, callus tissue had measurable signal in our AcBr lignin (79.4 mg lignin g^−1^ CW) assay and was most similar in quantity to seedling and expanding leaves.

The Bd21-3 stem lignin content increases during the development from expanding to mature developmental stages (Bd21-3: 12.8–15.8% CW; *p* < 0.05), consistent with previous observations for other monocot species [i.e., switchgrass (*Panicum virgatum*; 12 M sulfuric ADL; 7.5–8.6% NDF), big bluestem (*Andropogon gerardii*; 12 M sulfuric ADL; 6.8–7.4% NDF), tall fescue (*Festuca arundinacea* Schreb.; Klason lignin; 6.9–10.51% CW), maize (*Zea mays* L.; Klason lignin; 5–19% CW) and smooth brome (*Bromus inermis* Leyss.; AcBr lignin; 10.5–17% CW; Jung and Vogel, 1992; Chen et al., 2002; Casler and Hatfield, 2006; Jung and Casler, 2006]. Though numerical differences between Bd21-3 and Bd21 in measured lignin mean values for equivalent tissue and developmental stage existed, they were determined not to be statistically significant.

### HYDROXYCINNAMATES

**FIGURE 3:**
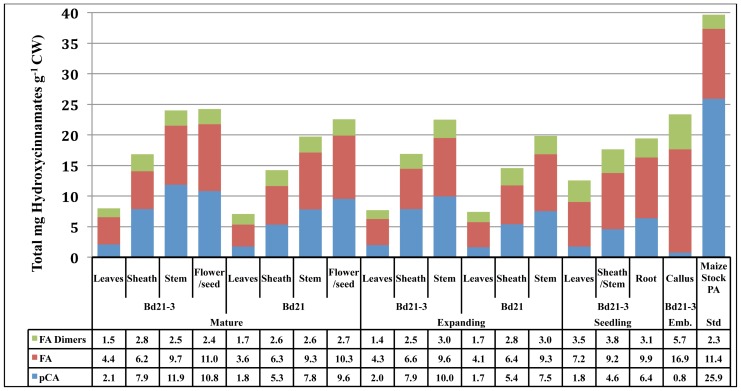
**Total phenolics (mg g^−1^ CW) of developing *Brachypodium* cell walls across developmental stage, accession, and tissue**. Blue, *pCA*; red, FA; green, the sum of all dimeric FA forms detected; Emb, embryonic.

The cell walls of *Brachypodium distachyon*, like other grasses, contain ester- and ether-linked non-lignified *p*-hydroxycinnamates (Harris and Hartley, 1980; Hatfield and Marita, 2010). Ferulic acid (FA) has been proposed to be critical in lignin polymerization initiation and cell wall crosslinking through various dimeric FA (DFA) moieties (Grabber et al., 1995; Ralph et al., 1995; Hatfield et al., 1999b). Though present in substantial amounts, *p*-coumaric acid (*p*CA) function in grass cell walls is unknown but has been postulated to have a role in radical transfer during lignification (Hatfield et al., 2008a,b, 2009; Ralph, 2010). Analysis of ether- and ester-linked phenolics was performed and compiled values for *Brachypodium distachyon* cell wall total FA, *p*CA, and DFA are presented (**Figure 3**).

Total phenolic content of *Brachypodium* cell walls significantly increase according to organ type, with leaves being the lowest, then sheaths and stems having the highest amounts. This trend for aerial organs holds within any developmental stage being analyzed. For expanding and mature organs, the cell walls of stems have approximately threefold more phenolics than do leaf organ cell walls. In mature plants, reproductive organ lignin content was statistically similar to stems. The total phenolic content of Bd21-3 stems at both expanding (22.6 mg total phenolics g^−1^ CW) and mature (24.1 mg total phenolics g^−1^ CW) developmental stages are significantly higher than Bd21 (19.8 and 19.7 mg total phenolics g^−1^ CW, respectively), thus indicating *Brachypodium* accession variability in cell wall composition.

Cell wall DFA content variation was primarily between leaves versus other organs. In expanding and mature developmental stages, leaf DFA content (1.4–1.7 mg total DFAs g^−1^ CW) was lower than both sheaths and stems (2.5–3.0 mg total DFAs g^−1^ CW). Interestingly, DFA content decreased in leaves on the developmental transition from 12-day-old seedlings (3.5 mg total DFAs g^−1^ CW) to our “expanding” stage (1.4 mg total DFAs g^−1^ CW). The biological significance of this DFA decrease is unknown.

Ferulic acid cell wall abundances vary primarily according to tissue with select developmental changes occurring but no significant variation between accessions being observed. In expanding and mature plants, FA content increased in level from leaves (3.6–4.1 mg total FAs g^−1^ CW) to sheaths (6.2–6.6 mg total FAs g^−1^ CW)) to stems (9.3–9.6 mg total FAs g^−1^ CW). Similar to DFAs, seedling leaf cell wall FA (7.2 mg total FA g^−1^ CW) is much higher than levels present at later developmental stages (4.3–4.4 mg total FA g^−1^ CW). Seedling sheath/stem tissue levels reflect more developmentally mature stem levels.

Total *p*CA cell wall content demonstrated significant variation according to organ, development and accession. Organ cell wall *p*CA content within each developmental stage was significantly different with the only exception being when stems were compared to the reproductive organs in mature plants. Cell wall *p*CA increased in levels moving from leaves to sheaths to stem. Developmentally, *p*CA levels increased from seedling (4.6 mg total *p*CA g^−1^ CW) to expanding plants (7.9–10 mg total *p*CA g^−1^ CW). In addition, Bd21-3 stem *p*CA increased from expanding (10 mg total *p*CA g^−1^ CW) to mature (11.9 mg total *p*CA g^−1^ CW) plants. Comparison of Bd21-3 and Bd21 stem *p*CA indicated a maintained and significant variation was present at both expanding and mature plants, with Bd21-3 (10 and 11.9 mg total *p*CA g^−1^ CW, respectively) having more than Bd21 (7.5 and 7.8 mg total *p*CA g^−1^ CW, respectively). These latter data indicate that the observed variation in total phenolics can be attributed to variation in *p*CA levels with Bd21-3 typically having more *p*CA incorporated into the cell walls when compared to Bd21.

*Brachypodium* callus cell wall phenolic composition was unique. Callus cell walls contained significantly higher levels of DFAs and FA than any other source analyzed in this study. In contrast, callus *p*CA was exceptionally low (0.8 mg total *p*CA g^−1^ CW). *p*CA has been identified as a substituent on arabinoxylans, but at levels significantly lower when compared to ferulates (Hartley et al., 1990). Grass cell wall *p*CA levels positively correlate with lignification (Hatfield et al., 2008b; Shen et al., 2009) and the ratio of *p*CA/lignin would expect to increase with *p*CA modification of lignin. *Brachypodium* callus *p*CA/lignin ratios are exceptionally low (0.01) while expanding and mature Bd21-3 ratios are much higher (0.03 for leaves, 0.06 for sheaths, and 0.08 for stems). Whether the apparent lignin observed in *Brachypodium* callus is real remains to be determined and may represent high levels of ferulates in the walls that appear to be lignin in the AcBr assay.

### URONIC ACIDS

Uronic acids, primarily glucuronic and galacturonic acids, are C6-oxidized forms of glucose (Glc) and galactose (Gal), respectively, and are components primarily of grass hemicelluloses (glucuronosyl arabinoxylans) and pectins (Carpita, 1996; Scheller and Ulvskov, 2010). *Brachypodium* organ cell wall total uronic acids ranged from 48 to 68 mg g^−1^ CW for Bd21 flowers/seeds to Bd21-3 embryonic callus, respectively (**Table 2**).

**Table 2:** Uronic acid content of *Brachypodium* cell walls*.

Developmental stage	Accession	Organ	Uronic acids
Mature	Bd21-3	Leaves	63.7 ± 7.7
		Sheath	63.5 ± 3.6
		Stem	57.7 ± 5.6
		Flowers/seed	50.1 ± 6.6
	Bd21	Leaves	61.3 ± 6.8
		Sheath	57.7 ± 6.6
		Stem	57.4 ± 5.2
		Flowers/seed	47.9 ± 4.2
Expanding	Bd21-3	Leaves	60.3 ± 8.6
		Sheath	60.1 ± 4.2
		Stem	60.3 ± 9.7
	Bd21	Leaves	61.3 ± 14.1
		Sheath	60.4 ± 11.3
		Stem	59.7 ± 10.1
Seedling	Bd21-3	Leaves	66.2 ± 0.3
		Sheath/stem	58.6 ± 1.5
		Roots	49.3 ± 0.6
Embryonic	Bd21-3	Callus	67.7 ± 1.7

* *mg g^−1^ CW ± standard deviation; averaged over two replicates*.

Statistical analysis indicated that total uronic acid content in *Brachypodium* cell walls did not vary regardless of tissue source, developmental maturity of the plant, nor accession analyzed (*p*-values ≥ 0.05).

### NEUTRAL SUGARS

Neutral sugars are principle component subunits of the plant cell wall structural polymers cellulose, hemicelluloses, and pectin (Carpita and McCann, 2000). The carbohydrate cell wall biopolymers are of special interest in both animal nutrition (National Research Council, 2001; Hatfield et al., 2007) and biofuel production (Carroll and Somerville, 2009). For energy conversion and biofuel production, the neutral sugars are the prime source for metabolic energy and thus bioavailability of these molecules directly relates to energy output. The neutral sugar profiles of cell walls prepared from developing *Brachypodium distachyon* organs were determined (**Table 3**).

**Table 3:** Neutral sugar content of *Brachypodium* cell walls*.

Developmental stage	Accession	Organ	Rhamnose	Fucose	Mannose	Galactose	Arabinose	Xylose	Glucose
Mature	Bd21-3	Leaves	2.9 ± 0.3	0.6 ± 0.2	1.4 ± 0.2	6.1 ± 1.1	20.2 ± 2.4	96.9 ± 3.2	240.4 ± 15.8
		Sheath	2.9 ± 0.0	0.6 ± 0.1	2.0 ± 0.1	6.5 ± 0.8	33.5 ± 0.8	204.0 ± 1.3	311.1 ± 3.4
		Stem	2.7 ± 0.1	1.2 ± 0.1	1.8 ± 0.2	4.9 ± 0.3	24.7 ± 1.2	212.4 ± 6.3	354.3 ± 17.2
		Flowers/seed	2.4 ± 0.0	0.6 ± 0.1	1.5 ± 0.2	5.4 ± 0.3	30.6 ± 0.6	245.0 ± 2.6	315.8 ± 9.4
	Bd21	Leaves	2.8 ± 0.2	0.4 ± 0.1	1.5 ± 0.1	5.9 ± 0.2	19.9 ± 0.5	89.4 ± 4.6	234.2 ± 5.3
		Sheath	2.8 ± 0.1	0.9 ± 0.2	2.0 ± 0.2	6.5 ± 0.2	32.6 ± 0.6	205.0 ± 6.4	327.3 ± 7.8
		Stem	2.7 ± 0.0	1.1 ± 0.1	1.6 ± 0.1	4.2 ± 0.1	22.8 ± 0.2	212.7 ± 1.9	379.7 ± 4.7
		Flowers/seed	2.4 ± 0.0	0.8 ± 0.0	1.3 ± 0.1	5.0 ± 0.2	29.0 ± 0.5	246.6 ± 15.7	322.2 ± 1.0
Expanding	Bd21-3	Leaves	2.8 ± 0.3	0.4 ± 0.0	1.5 ± 0.2	4.9 ± 0.4	20.8 ± 5.8	103.7 ± 33.8	255.2 ± 43.9
		Sheath	2.7 ± 0.1	0.6 ± 0.3	2.1 ± 0.3	6.9 ± 1.7	33.4 ± 4.6	213.7 ± 30.2	317.2 ± 35.5
		Stem	2.8 ± 0.0	0.7 ± 0.7	2.4 ± 0.0	6.3 ± 0.7	30.4 ± 1.4	236.1 ± 24.2	347.6 ± 22.1
	Bd21	Leaves	2.8 ± 0.4	0.4 ± 0.0	1.7 ± 0.4	5.7 ± 1.4	21.0 ± 6.4	100.5 ± 30.0	268.6 ± 55.8
		Sheath	2.8 ± 0.1	0.5 ± 0.3	2.3 ± 0.3	7.1 ± 1.9	35.3 ± 4.6	222.3 ± 20.9	338.7 ± 23.5
		Stem	2.8 ± 0.0	0.7 ± 0.3	2.1 ± 0.3	6.0 ± 1.8	29.0 ± 4.0	239.8 ± 6.0	362.2 ± 7.0
Seedling	Bd21-3	Leaves	2.9 ± 0.0	0.3 ± 0.0	2.0 ± 0.1	5.0 ± 3.4	27.0 ± 2.4	111.9 ± 7.2	293.8 ± 4.5
		Sheath/stem	2.7 ± 0.1	0.4 ± 0.0	3.4 ± 0.1	15.7 ± 1.4	42.9 ± 0.4	159.5 ± 3.0	297.0 ± 4.0
		Roots	2.7 ± 0.2	0.6 ± 0.1	4.1 ± 0.3	34.3 ± 1.8	46.2 ± 2.5	160.4 ± 2.7	291.6 ± 5.0
Embryonic	Bd21-3	Callus	3.7 ± 0.1	0.8 ± 0.0	2.7 ± 0.1	56.3 ± 2.1	94.3 ± 2.4	115.5 ± 0.5	122.3 ± 1.1

* mg g^−1^ CW ± standard deviation; averaged over two replicates.

The carbohydrates can be divided into two groups based on abundance: (1) major, including arabinose (Ara), xylose (Xyl), Glc, and Gal, and (2) minor, including rhamnose (Rha), fucose (Fuc), and mannose (Man).

Glucose, the principle component of cellulose and the mixed-linkage β-glucan and xyloglucan, is the most abundant cell wall neutral sugar throughout development. Absolute Glc cell wall abundance primarily varied according to organ in expanding and mature plants. Glc levels were lowest in leaves and highest in stems. In seedlings, Glc cell wall abundance did not vary between organs. Callus cell wall Glc was significantly lower than all other cell walls analyzed. An alternative approach to view sugar content is to calculate the molar percentage of individual sugars relative the total neutral sugar content (**Table 4**). As a molar percentage, Glc comprised approximately 62% of leaf, 51% of sheath, and 54% of stem neutral sugars. However, callus walls were an exception where Glc made up only 27.9% of the total cell wall sugar.

**Table 4:** Major neutral sugar mole percentage in *Brachypodium* cell walls*.

Developmental stage	Accession	Organ	Glucose	Xylose	Arabinose	Galactose	Total
Mature	Bd21-3	Leaves	61.3	29.6	6.2	1.6	98.7
		Sheath	51.1	40.2	6.6	1.1	99.0
		Stem	54.5	39.2	4.6	0.7	99.1
		Flowers/seed	48.1	44.8	5.6	0.8	99.3
	Bd21	Leaves	62.2	28.5	6.3	1.6	98.6
		Sheath	52.4	39.4	6.3	1.0	99.0
		Stem	56.5	38.0	4.1	0.6	99.1
		Flowers/seed	48.6	44.6	5.2	0.8	99.3
Expanding	Bd21-3	Leaves	61.6	30.0	6.0	1.2	98.8
		Sheath	50.6	40.9	6.4	1.1	99.1
		Stem	51.1	41.7	5.4	0.9	99.1
	Bd21	Leaves	63.2	28.4	5.9	1.3	98.8
		Sheath	51.3	40.4	6.4	1.1	99.1
		Stem	52.0	41.3	5.0	0.9	99.1
Seedling	Bd21-3	Leaves	62.4	28.5	6.9	1.1	98.8
		Sheath/stem	52.8	34.0	9.1	2.8	98.8
		Roots	50.2	33.1	9.5	5.9	98.7
Embryonic	Bd21-3	Callus	27.9	31.6	25.8	12.9	98.3

* Mole percentage of individual sugar to total neutral sugar amount from organ cell wall.

The grass cell wall biopolymer sources of Xyl and Ara are primarily arabinoxylans but xyloglucans, arabinogalactans, and possibly even arabinogalactan proteins could contribute (Carpita, 1996; Seifert and Roberts, 2007; Scheller and Ulvskov, 2010). Xyl and Ara were the second and third, respectively, most abundant neutral sugars present in developing *Brachypodium* cell walls. Xyl amounts ranged in absolute amounts of 89.4–246.6 mg g^−1^ CW (**Table 3**) and mole percentage of 28.4–44.8 (**Table 4**). Regardless of developmental stage and accession, Xyl amounts primarily varied according to organ with leaf cell walls being significantly lower than the other organs. Organ-specific abundance of Xyl complemented the Glc profile; a lower Glc amount correlated with higher Xyl. Callus levels of Xyl were near equivalent to those obtained for seedling-derived walls, which would consist of primary cell walls. In expanding and mature developmental stages, cell wall Ara was highest it sheaths and lowest in leaves (**Table 3**). Though absolute abundance varied according to organ, the Ara cell wall mole percentages were equivalent for leaf and sheath and lower for stems. However, callus (25.8 mol%) derived cell walls had much higher amounts of Ara. In general, Xyl represents the second most abundant *Brachypodium* cell wall carbohydrate behind Glc. However, an exception was observed with *Brachypodium* callus cell walls where Xyl is the most abundant carbohydrate based on mole percentage present (**Table 4**).

The cell wall molar ratio of Ara to Xyl and FA to Ara can be used as indicators of the degree of Ara modification of xylans and the degree of FA modification of arabinoxylans that is implicit in the degree of cell wall cross-linking (Carpita, 1996). Cell wall molar ratios of Ara to Xyl and FA to Ara are presented (**Table 5**).

**Table 5:** Ara/Xyl and FA/Ara cell wall molar ratios.

Developmental stage	Accession	Organ	Ara/Xyl	FA/Ara
Mature	Bd21-3	Leaves	0.21	0.17
		Sheath	0.16	0.14
		Stem	0.12	0.30
		Flower/seed	0.12	0.28
	Bd21	Leaves	0.22	0.14
		Sheath	0.16	0.15
		Stem	0.11	0.32
		Flower/seed	0.12	0.28
Expanding	Bd21-3	Leaves	0.20	0.16
		Sheath	0.16	0.15
		Stem	0.13	0.24
	Bd21	Leaves	0.21	0.15
		Sheath	0.16	0.14
		Stem	0.12	0.25
Seedling	Bd21-3	Leaves	0.24	0.21
		Sheath/stem	0.27	0.17
		Root	0.29	0.17
Embryonic	Bd21-3	Callus	0.82	0.14

Molar ratios of Ara to Xyl in developed organs were highest in leaves and lowest in stems. Whereas, the FA to Ara ratio was highest in more mature stems and approximately the same in either sheath or leaf cell walls. These data are consistent with having higher levels of crosslinking in more mature stems with a greater percentage of the Ara units modified with FA.

Grass cell wall associated Gal is predominantly derived from galactans and arabinogalactans (Carpita, 1996). During *Brachypodium* development, Gal content of cell walls is higher during early development (i.e., seedling) and in undifferentiated callus rather than in later developmental stages (**Table 3**) as expected for developmental stages with more primary cell wall. Statistical analysis of cell wall Gal abundance and mole percentages indicate that little variation occurs in aerial organs throughout later stages of development. The primary organ cell wall variation is observed between seedling organs where leaves, stem/sheaths, and root are all different. In addition, callus cell wall Gal content is the highest of any measured in this study and, when translated into mole percentage, represents a major sugar of callus cell walls. Overall, Gal cell wall abundance correlates well with organs/cells containing predominantly primary cell wall (Carpita, 1996).

The abundance of the minor sugars Rha, Fuc, and Man combined did not exceed 2 mol% throughout development. Rha cell wall content does not vary in *Brachypodium* tissues throughout our entire plant tissue series. The only statistically significant difference was higher levels in callus cell walls compared to other samples. Fuc did not exceed 0.19 mol% and did not significantly change in abundance between any of our samples. Man did not exceed 0.7 mol% present in seedling roots. Seedling root cell wall Man was significantly higher than all other cell walls except seedling sheath/stem and callus. Though important biologically, Rha, Fuc, and Man are minor cell wall sugars throughout developing *Brachypodium *organs.

### TOTAL CELL WALL COMPOSITION

**FIGURE 4:**
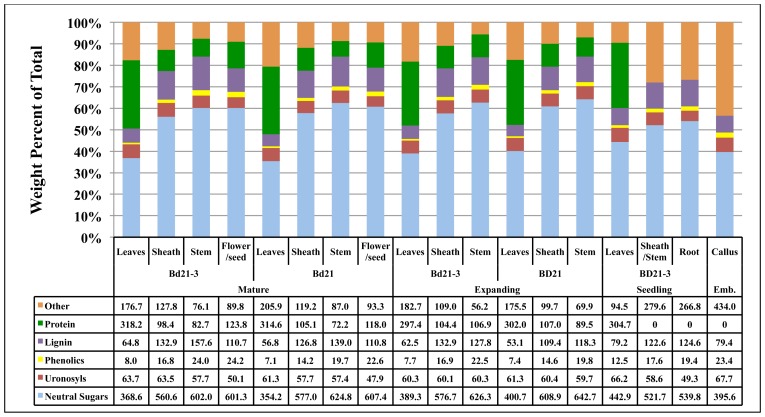
**Contribution of *Brachypodium *cell wall components to the total cell wall composition**. The consolidation of cell wall weight percentages of neutral sugars (light blue), uronosyls (red), phenolics (yellow), lignin (purple), protein (green), and other (tan) to equal 100% is given for each tissue from each developmental stage. Note: protein analysis of seedling sheath/stem, root, and embryonic callus were not performed and therefore “other” includes protein for these tissues. Numbers in table are in the units of mg component g^−1^ cell wall. Emb, embryonic.

The total compositions of developing *Brachypodium* cell walls were determined (**Figure 4**).

To facilitate this, remaining protein content for isolated cell walls from composite plant materials at developmental stages 1 and 2 were determined and then weight percentages compiled based on general categories including neutral sugars, uronosyls, phenolics, lignin, protein, and “other.” The “other” category includes presumably cutins, suberins, and other hydrophobic compounds associated with the organ cell walls (Carpita, 1996). Our total cell wall analysis was performed on individual cell wall preparations to minimize cell wall batch variation and, as a result, insufficient quantities of sample were available to perform protein assays on Bd21-3 seedling sheath/stem, seedling root, and callus cell walls. Therefore, a component of the “other” category for these samples will be protein. Overall, the primary variation in cell wall composition was attributed more to plant organ type rather than developmental stage of the plant. The alterations in the neutral sugar, protein, and lignin content were the primary sources of compositional variation. Associated protein was a much higher relative component of leaf cell walls compared to sheath or stem. Conversely, neutral sugar and lignin proportions of the cell walls increased significantly in sheath and even more so in stem. Though variations were observed in lignin and phenolic content, the difference in cell wall compositions between accessions Bd21 and Bd21-3 was minor. Overall, composition analysis of cell walls derived from distinct organs of developing *Brachypodium* indicates that the greatest deviation between samples occurs comparing organ-specific wall composition independent of stage of maturation.

**FIGURE 5:**
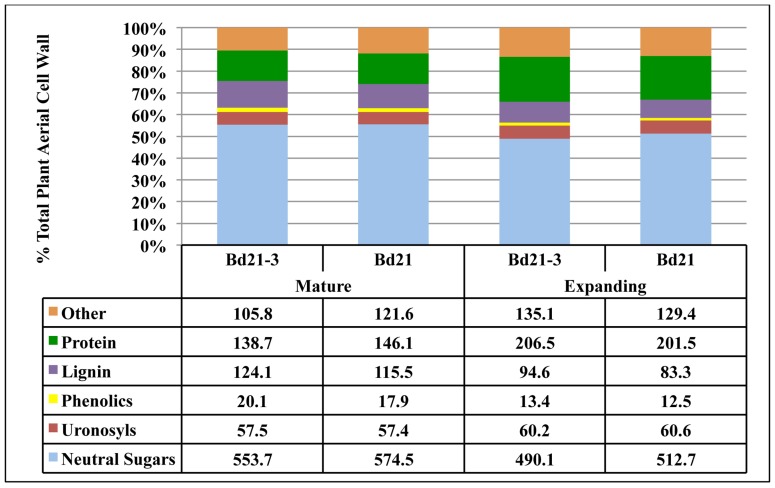
**Determination of total aerial plant cell wall composition for *Brachypodium distachyon*.** Using total tissue harvest yields, tissue cell wall yields and tissue compositional analysis, values were calculated to determine expected cell wall composition if whole aerial portions of Brachypodium were used as input for cell wall isolation and analysis. Numbers in table are in the units of mg component g^−1^ cell wall.

Due to the small stature of *Brachypodium distachyon* and low organ yields per plant, it was prohibitive to analyze distinct organs from individual plants. Therefore, using our compiled results for mean organ fresh weight harvested (**Table 6**) and mean cell wall yields per organ type (**Table 7**), we calculated the expected composition of cell walls if derived from harvests of the total aerial portions of Bd21 or Bd21-3 plants at either mature or expanding developmental stages (**Figure 5**).

**Table 6:** Relative distribution of aerial plant organs based on harvest fresh weight*.

Developmental stage	Accession	Organ	% Aerial fresh weight
Mature	Bd21-3	Leaves	22.5 ± 9.4
		Sheath	13.7 ± 1.1
		Stem	42.0 ± 5.1
		Flowers/seed	21.9 ± 5.4
	Bd21	Leaves	25.1 ± 2.4
		Sheath	8.7 ± 0.1
		Stem	41.0 ± 0.5
		Flowers/seed	25.3 ± 1.8
Expanding	Bd21-3	Leaves	52.0 ± 0.8
		Sheath	19.1 ± 0.5
		Stem	28.9 ± 1.2
	Bd21	Leaves	53.5 ± 8.1
		Sheath	15.2 ± 1.5
		Stem	31.2 ± 6.6

* Mean percentage of specific organ weight divided by total weight of all organ harvested for accession at developmental stage ± value range from mean.

**Table 7:** Relative contribution of cell wall material per aerial organ*.

Developmental stage	Accession	Organ	% Cell wall
Mature	Bd21-3	Leaves	17.9 ± 7.9
		Sheath	15.3 ± 1.7
		Stem	38.9 ± 3.5
		Flowers/seed	27.9 ± 6.1
	Bd21	Leaves	22.6 ± 5.3
		Sheath	9.1 ± 0.0
		Stem	41.8 ± 0.2
		Flowers/seed	29.7 ± 1.8
Expanding	Bd21-3	Leaves	52.6 ± 0.6
		Sheath	23.4 ± 0.5
		Stem	24.0 ± 1.1
	Bd21	Leaves	51.2 ± 8.0
		Sheath	18.4 ± 1.7
		Stem	30.4 ± 6.3

* Mean percentage of specific organ cell wall material to total aerial cell wall for accession and developmental stage ± value range from mean; means used: expanding: leaves: 51.9, sheath: 20.9, and stem 27.2; mature: leaves: 20.3, sheath: 12.2, stem: 40.3, and flowers/seed: 28.8.

## DISCUSSION

*Brachypodium distachyon* has recently been championed as a model system for cereals and forage grasses. Significant progress has been made to characterize genomic and transcriptomic attributes of *Brachypodium* accessions while developing tools to facilitate functional genomics research. A major impetus for this rapid movement is to fulfill a need for a grass model system. Though given the immense impact of *Arabidopsis thaliana* in general plant biology and specifically with dicots, it is recognized that *Arabidopsis* is not an appropriate model for all aspects of monocot biology. *Brachypodium distachyon* clearly has physical and genetic attributes that make it a desirable grass model system and much credence has been based on the phylogenetic relationship that *Brachypodium distachyon* shares with temperate cereal and forage grasses. An important next-step to this work is to validate experimentally that *Brachypodium* does indeed have the physical and chemical attributes to make it an appropriate model for forage grasses.

Forage grasses are used typically for animal feed and are harvested and stored at developmental stages when organs have significant levels of secondary cell wall. Presumably to facilitate yields, these same stages would be used as feedstock for biofuel. The diversity of grasses used for forage do differ in their cell wall compositions (Hatfield et al., 2009). Therefore, a need exists to validate whether *Brachypodium distachyon* is a suitable model for forage grass cell walls at mature plant developmental stages. We have taken an analytical chemical approach to determine the organ-specific cell wall compositions of diploid *Brachypodium* accessions through four stages of plant development. The developmental stages included two agronomically relevant advanced stages: (1) “expanding” – transition of vegetative-to-reproductive growth, and (2) “mature” – seed fill, and two stages to support laboratory research agendas: (3) embryonic callus and (4) 12-day-old seedlings.

Our work complements three previous studies analyzing cell wall compositions in *Brachypodium distachyon* (Christensen et al., 2010; Guillon et al., 2011; Opanowicz et al., 2011). The work of Opanowicz et al. (2011) and Guillon et al. (2011) focused on developmental changes during grain maturation and grain cell wall compositions in *Brachypodium distachyon*. When compared to cereals, Opanowicz et al. (2011) noted that *Brachypodium* endosperm contains thick cell walls. Using FT-IR imaging, they suggested that the cell walls contained (1,3;1,4)-β-D-glucan and arabinoxylans at levels similar to barley and oats. The work of Guillon et al. (2011) used a combination of chemical analysis, enzymatic fingerprinting, and immune-localization-based imaging to localize and characterize the cell walls of the *Brachypodium* grain. Comparison of the our results to Guillon et al. (2011) indicate that vegetative cell walls have drastically more Xyl (~90–245 mg g^−1^ CW versus 14–37 mg g^−1^ CW) and less Glc (~234–380 mg g^−1^ CW versus 548–624 mg g^−1^ CW) than do grain cell walls. In addition, phenolic content of the respective cell walls were different with vegetative tissues having much higher levels of total hydroxycinnamates and the relative contribution of *p*CA and DFAs being much more. The work of Christensen et al. (2010) focused on comparing growth characteristics with hydroxycinnamate and hemicellulose [esp. (1,3;1,4)-β-D-glucan and arabinoxylans] content of cell walls between *Brachypodium distachyon*, barley (*Hordeum vulgare* L.), and wheat (*Triticum aestivum* L.) during early seedling development (days 3–8 post-germination). Their approach relied on collection of entire plants over a relatively short time-course for cell wall preparations and chemical analysis on those AIR preparations. Their carbohydrate analysis involved mild TFA hydrolysis to release “non-cellulosic” neutral sugars for analysis. For our approach, we were interested in identifying organ and developmental variations in total cell wall composition over a much larger developmental time span with special consideration on later, more agriculturally pertinent developmental stages. In addition, we isolated specific plant organs at these distinct developmental stages and prepared organ-specific cell wall preparations for each to allow for comparative analyses to be made. Therefore, interested in total cell wall carbohydrate composition, we chose to use a strong acid hydrolysis to liberate all carbohydrates from our cell wall preparations. With these caveats of differing experimental approaches, only limited comparison can be made between the two data sets. Analysis in Christensen et al. (2010) of mole percentage of Ara and Xyl from *Brachypodium* seedling cell walls suggested a trend of a decreasing Ara/Xyl ratio (day 3: 0.38 to day 8: 0.30). Our data for 12-day-old seedlings show a Ara/Xyl molar ratio to be consistent with this trend (leaves: 0.24, sheath/stem: 0.27, and roots: 0.29). For more mature tissues, this ratio continues down to around 0.21 to 0.12, suggesting that the Ara modification of xylans possibly decreases with plant maturity. In addition, Christensen et al. (2010) analyzed ester-linked hydroxycinnamates via the mild saponification and HPLC approach of Waldron et al. (1996) compared to our updated approach of total saponification and GC analysis. Comparison of results indicated a large difference in levels of hydroxycinnamates detected with Christensen et al. (2010) reporting 0.9 mg total ester-linked hydroxycinnamates g^−1^ AIR while our seedling results ranged from 12.5 to 19.4 mg total phenolics g^−1^ CW of which approximately 78% was ester-linked. The undetermined cause of the large difference could be attributed to method variation in cell wall/AIR preparations and/or hydroxycinnamate analytical procedures.

Recent work by Shen et al. (2009) looked at lignification and hydroxycinnamate content of switchgrass during development showing a negative relationship with biomass saccharification efficiency. At comparable developmental stages, *Brachypodium* cell wall AcBr lignin content was lower than that measure in switchgrass cell walls. Switchgrass lignin abundance was approximately 3.2-fold higher in leaves, 1.6-fold higher in sheaths, and two-fold higher in stems than what was measured here for *Brachypodium distachyon*. However, hydroxycinnamate content of *Brachypodium* cell walls appears to be higher that that observed for switchgrass. When using expanding Bd21-3 for comparison to E4-I2 switchgrass values from Shen et al. (2009), *Brachypodium* stem cell walls have comparable *p*CA values (10.0 versus ~10.7 mg g^−1^ CW for switchgrass) and higher FA amounts (9.6 versus ~4.6 mg g^−1^ CW for switchgrass). These results suggest that *Brachypodium distachyon* may be useful to model cell wall properties for bioenergy crops.

The overall organ-specific cell wall chemical compositions from *Brachypodium distanchyon* were similar to those previously determined for a diverse set of C3 forage grasses, including tall fescue (*Festuca arundinacea *Schreb.), bromegrass (*Bromus inermis* Leyss.), orchardgrass (*Dactylis glomerata* L.), reed canarygrass (*Phalaris arundinacea* L.), winter wheat (*Triticum aestivum* L.), and oats (*Avena sativa* L.), developmentally equivalent to our “expanding” stage (Hatfield et al., 2009). Ester-linked *p*CA and FA abundance in stem cell walls for *Brachypodium* matched well with those determined for orchardgrass. In addition, Gal and Man concentrations did not deviate much at 6.2 versus 6.1, and 2.3 versus 2.0 mg sugar g^−1^ cell wall, respectively for *Brachypodium* versus mean C3 forage grass. Though *Brachypodium* stems deviated from C3 grasses in the cell wall abundances of non-Glc neutral sugars, the variance of values for the C3 grasses made the differences statistically insignificant. For example, Xyl stem cell wall concentrations were elevated with *Brachypodium* cell walls containing a mean of 237 mg Xyl g^−1^ CW and other C3 grasses containing a mean of 173 mg Xyl g^−1^ CW. The variance of values for the C3 grasses made this difference statistically insignificant (*p* = 0.12).

A significant deviation between *Brachypodium* and other C3 forage grasses was observed in the stem lignin and Glc content. Forage grass stem lignin concentrations ranged from 160 to 210 mg lignin g^−1^ cell wall with a mean of approximately 193 mg lignin g^−1^ cell wall. The *Brachypodium* mean value for the similar developmental stage was 123 mg lignin g^−1^ cell wall. Conversely, the Glc concentration was increased in *Brachypodium* stem cell walls, with mean values for Glc at 355 mg g^−1^ cell wall being much higher than the C3 grass mean of 240 mg Glc g^−1^ cell wall (range 158.9–319.4 mg Glc g^−1^ cell wall; *p* = 0.00038). These differing values represent relative increases in *Brachypodium* stem cell walls for Glc of 48% and a decrease in lignin of 36%. One explanation for the discrepancy of stem cell wall composition would be that *Brachypodium*, due to its small stature, has evolved not to need lignin to the same extent as the larger C3 grasses for structural support. Therefore, carbohydrate polymer replacement for lignin might be sufficient for it to reach its full developmental stature.

The cell wall composition profiles in embryonic callus were significantly different from later developmental stages. The most notable changes were the relative increase in mole percentage of Gal and Ara at the expense of Glc (**Table 4**). The most likely source of these sugars in primary grass cell walls is arabinogalactans (Carpita, 1996) and arabinogalactan proteins thus suggesting a significant role of pectins in undifferentiated *Brachypodium* tissue. In addition, total callus cell wall FA is the highest and *p*CA content the lowest of any developmental stage in *Brachypodium*. It has been demonstrated that undifferentiated grass cell lines containing primary cell walls possess the capacity to lignify when supplied with exogenous monolignols and H_2_O_2_ substrate for peroxidase activity (i.e., Grabber et al., 1995). Due to proposed roles for each (Ralph, 2010), it is consistent that the primary cell walls of the *Brachypodium* callus is establishing the foundation for future lignification without completing it.

Overall, the organ-specific cell wall developmental analysis presented here establishes a baseline for comparison of this putative model forage grass with established, agronomical important crop plants. This work also provides a reference from which future genetic manipulation of cell wall composition can be compared. Though differences do exist between *Brachypodium* and other C3 grass cell wall compositions, we believe that *Brachypodium* can be a useful model for forage grass research.

## Conflict of Interest Statement

The authors declare that the research was conducted in the absence of any commercial or financial relationships that could be construed as a potential conflict of interest. Mention of a trademark or proprietary product does not constitute a guarantee or warranty of product by the USDA and does not imply its approval to the exclusion of other products that may also be suitable.

## Abbreviations

AcBr: acetyl bromide
Ara: arabinose
BBCH-scale: Biologische **B**undesanstalt **B**undessortenamt und **CH**emische Industrie plant developmental scale
CW: cell wall
DFA: dehydrodiferulic acid
FA: ferulic acid
Fuc: fucose
Gal: galactose
Glc: glucose
Man: mannose
*p*CA: *p*-coumaric acid
Rha: rhamnose
Xyl: xylose.
